# Perspectives for Synergic Blends of Attractive Sources in South American Palm Weevil Mass Trapping: Waiting for the Red Palm Weevil Brazil Invasion

**DOI:** 10.3390/insects12090828

**Published:** 2021-09-14

**Authors:** Viviane Araujo Dalbon, Juan Pablo Molina Acevedo, Karlos Antônio Lisboa Ribeiro Junior, Thyago Fernando Lisboa Ribeiro, Joao Manoel da Silva, Henrique Goulart Fonseca, Antônio Euzébio Goulart Santana, Francesco Porcelli

**Affiliations:** 1 Natural Resources Research Laboratory, Center for Engineering and Agrarian Sciences, Federal University of Alagoas, Renorbio (LPqRN, CECA, Renorbio-UFAL), Av. Lourival Melo Mota, S/N, Tabuleiro do Martins, Maceió 57072-900, AL, Brazil; karloslisboa@gmail.com (K.A.L.R.J.); tflribeiro@gmail.com (T.F.L.R.); fonsecagoulart@gmail.com (H.G.F.); aegs@ceca.ufal.br (A.E.G.S.); 2 Colombian Corporation for Agricultural Research Agrosavia C. I. Turipana—AGROSAVIA, Km. 13, Vía Montería-Cereté 230558, Córdoba, Colombia; juanpamolina@yahoo.com.br; 3 Microbiology Research Laboratory, Center for Engineering and Agrarian Sciences, Federal University of Alagoas, Renorbio (LM, CECA, Renorbio-UFAL), Av. Lourival Melo Mota, S/N, Tabuleiro do Martins, Maceió 57072-900, AL, Brazil; jm.agro@hotmail.com; 4 Dipartimento di Scienze del Suolo, della Pianta e degli Alimenti, University of Bari Aldo Moro, Via Amendola, 165/a, 70126 Bari, Italy; francesco.porcelli@uniba.it; 5 CIHEAM Mediterranean Agronomic Institute of Bari, Via Ceglie 9, 70010 Bari, Italy

**Keywords:** preventive and protective alien invasive and quarantine pest IPM, *Dynamis borassi*, *Cosmopolites sordidus*, banana weevil

## Abstract

**Simple Summary:**

Palm weevils—both South American and red palm weevils—threaten economically relevant palms, affecting oil and fruit production with a corresponding social impact. The natural tendency of the red palm weevil to explore vast territories in combination with corridors of cultivated susceptible palm species drives pest expansion in new territories. Invasion still occurs westward, from Sundaland to Portugal and the West-African coast, including the Canary Archipelago. The red palm weevil menaces the South American oil palm plantations, opening a new double-pest scenario and consequential damages or a possible coexistence with reciprocal competitive exclusion phenomena opportunities. On the brink of the red palm weevil’s entrance into South America, we present available options for multiple lure-use in contaminating stations for the sustainable and effective management of both pests.

**Abstract:**

Coupling several natural and synthetic lures with aggregation pheromones from the palm weevils *Rhynchophorus palmarum* and *R. ferrugineus* reveals a synergy that results in an increase in pest captures. The combined attraction of pure pheromones, ethyl acetate, and decaying sweet and starchy plant tissue increases the net total of mass-trapped weevils. The 2018 entrance of the red palm weevil (RPW) into South America has threatened palm-product income in Brazil and other neighboring countries. The presence of the new A1 quarantine pest necessitates the review of all available options for a sustainable mass-trapping, monitoring, and control strategy to ultimately target both weevils with the same device. The effective lure-blend set for the mass-trapping system will attract weevils in baiting and contaminating stations for entomopathogenic fungi that the same weevils will spread.

## 1. Introduction

The South American palm weevil (SAPW), *Rhynchophorus palmarum* (Linneaus 1764), and the red palm weevil (RPW), *Rhynchophorus ferrugineus* (Oliver, 1790), are well-known, relevant pest species worldwide [1,2,3,4,5,6,7,8,9,10,11,12]. Today, SAPW and RPW are the key invasive pests [13,14] of cultivated palms (Arecales: Arecaceae) in the old and new world. The two insects are Curculionidae (Coleoptera), included in the genus *Rhynchophorus* in a ten-species natural assemblage [15]. Eight *Rhynchophorus* species are considered pests because they damage fruit bunches and sometimes even kill the host palm [16]. Specifically, *R. ferrugineus* is a major threat to coconut production in Brazil [2,17]. The RPW has arrived in the Caribbean islands Aruba and Curaçao, near South America [10], and Brazil reports this insect as an A1 quarantine pest [2].

The invasive pest *R. ferrugineus* is responsible for killing thousands of ornamental Canary date palms (*Phoenix canariensis* Chabaud), first detected in San Diego County in Southern California, USA, in 2011 and established around 2010 [16]. Considered a worldwide pest, the related species *R. vulneratus* was declared eradicated from California [18]. Therefore, it is necessary to adopt multiple complementary tactics, including cultural measures, biological control, and semiochemicals, for an integrated pest management program (IPM) of *R. palmarum* and *R. ferrugineus* [19,20].

This review aims to inform and contribute to a better knowledge of the bait sources used to capture *R. palmarum* and, therefore, to facilitate adequate management of the *R. ferrugineus* quarantine pest. Here, the focus is on the efficacy of attractive sources and further pest control strategies for *R. palmarum* and *R. ferrugineus* in Arecaceae.

## 2. The Genus *Rhynchophorus* spp.

The *Rhynchophorus* genus includes oligophagous insect pests, which reproduce on a diverse variety of palm species (Arecales: Arecaceae) [15] with a complete metamorphosis, including the egg, larva, pupa, and adult stages [21]. In adulthood, males release the aggregation pheromone to attract males and females to a new food source when they reach a suitable host plant [22,23,24,25]. Females dig an egg chamber at the base of the spine or on a wounded plant part, and a female can lay approximately 250 eggs in many different palms during her life. The *Rhynchophorus* life cycle ranges from 132 to 215 days [26,27,28,29].

Morphologically, within the genus *Rhynchophorus*, chromatic polymorphism exists among the specimens [15,30,31,32]. Such variations may relate to environmental conditions, food availability, and plant lifecycle. We need further studies to identify the genetic and phylogenetic variations among populations and to characterize differences among invading insect populations [33].

Species in the genus exhibit behaviors and characteristic activity periods. For example, *R. palmarum* is diurnal with a maximum flight distance of 1.6 km in 24 h, regardless of sex. The more significant bioactivity is between the hours 9–11 and 17–19 within that period, decreasing in rainy periods [34,35]. The flight capabilities of *R. palmarum* in 24 h flight mill trials recorded average distances of 41 km (M) and 53 km (F) in 24 h in predominantly daytime [7,8], using field-collected male and female weevils. For *R. ferrugineus*, catches were higher between the hours of 8:00 and 18:00 [4,34] with the capacity to make long-distance flights reaching 7 km, corresponding to 3 to 5 days. However, for *Rhynchophorus* spp., biogeographic location conditions pest biology, as the species show extended biological plasticity, inflicting damage in different situations [36].

It is also characteristic of this genus that the larvae feed boring galleries into the Arecaceae stipes, destroying the palm apical meristem [4]. Given that *Rhynchophorus* has a palm borer lifestyle, the damage is based on larvae survivorship in the host palm trunk coupled with late damage and infestation diagnosis [17]. Thus, understanding the insect’s behavior is important for the implementation of new control strategies, especially those derived from research on telemetry [37], semiochemical interference [38,39,40,41], new secondary metabolites and formulations [42,43,44,45,46,47,48,49], early infestation detection [50], novel biopesticides [51,52,53,54], and natural enemies [55,56].

### 2.1. South American Palm Weevil

The pest *R. palmarum,* known as palm weevil or palm’s eye bit, oviposits in plants of the Arecaceae family. It is the primary pest in coconut (*Cocos nucifera* L.) and oil palm (*Elaeis guineensis* Jacq.) plantations. The New World distribution of *R. palmarum* encompasses the American continent, from Argentina to California, and includes the Central American Antilles [57].

**Figure 1:**
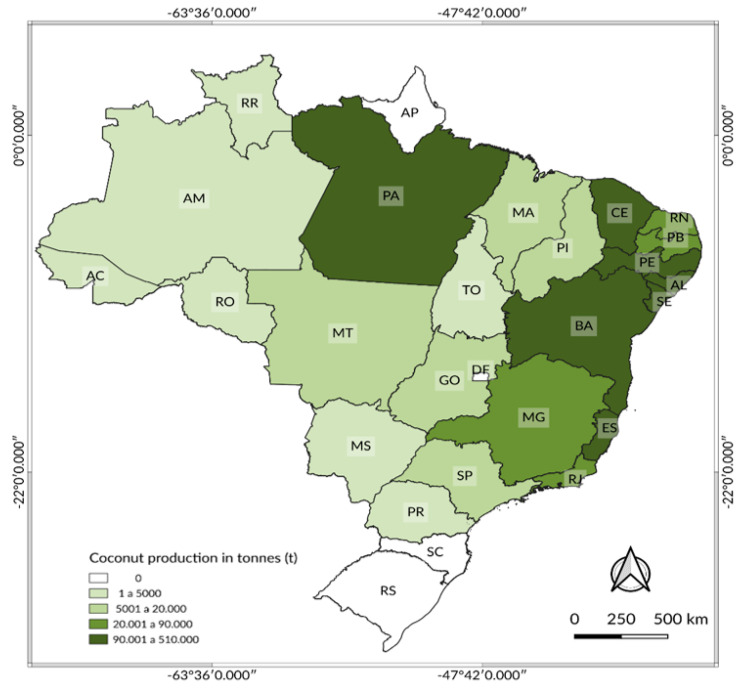
Coconut production map in Brazil; geodetic reference system (SIRGAS-2000).

The SAPW is a well-known plague of coconut palm and oil palm in Brazil, and the weevil has been reported in all the Brazilian areas of commercial palm production, where it causes relevant damages [58]. All states of Brazil such as Acre (AC), Alagoas (AL), Amazonas (AM), Bahia (BA), Ceará (CE), Espirito Santo (ES), Goiás (GO), Maranhão (MA), Mato Grosso (MT), Mato Grosso do Sul (MS), Minas Gerais (MG), Pará (PA), Paraíba (PB), Paraná (PR), Pernambuco (PE), Piauí (PI), Rio de Janeiro (RJ), Rio Grande do Norte (RN), Rondonia (RO), Roraima (RR), Sao Paulo (SP), Sergipe (SE), and Tocantins (TO) are particularly interested with the weevil outbreak and are the largest commercial coconut producers in ton (t) of Brazil (Figure 1).

#### Biology

The lifecycle of *R. palmarum* in palms completes in approximately 127 days, consisting of the expected holometabolic sequence of eggs, larvae, pupae, and adults (Table 1) [27,35].

Eggs: Female *R. palmarum* deposits, approximately some millimeters inside soft plant tissue white and elongate ovoid eggs long about 2.5 mm and large 1 mm, lasting 2–5 days [21,59] before hatching.

Larvae: The larvae *R. palmarum* are eucephalous, “C”-shaped, and apodous. Grubs are 75 mm long, creamy-white, and yellowing at the end of their instars stage. The larvae stage is between 45 and 70 days [21]. *Rhynchophorus palmarum* populations take advantage of the larvae’s cryptic habits as their growth occurs in the galleries formed within the plant, protecting them from possible natural enemies. It is also the vector responsible for transmitting the nematode *Bursaphelenchus cocophilus* (Cobb) Baujard (Parasitaphelenchidae), the causative agent of the red-ring-like disease in palms, with a unique record of increasing symptoms damage to palms only in America [60,61,62,63].

Pupae: The pupae *R. palmarum* are yellowish and remain housed within their 80–100 mm long pupal case that larvae set with coconut fibers, and the pupa stage has a variation of 25 to 45 days [21,27]. Adults: The black palm weevil measures between 35 mm and 60 mm, and the rostrum may serve to discriminate between male and female: the male snout is straight, stout, and shows series of stout seta set as in a brush on the front-clypeal head region, while the female rostrum is slender, devoid of seta, and slightly dorso-ventrally arched [15,21]. SAPW are active daytime in the morning and during the spring with warmer temperatures. Adults can fly up to 1.6 km per day [7,8], but it is not common to see them flying during the hottest hours of the day. Experience suggests that *R. palmarum* populations fluctuate in economic palm-intensive cultivation [64,65,66].

In adults, it has atypical chromatic natural polymorphism and colors with a different gradient of red in the cuticle in some *R. palmarum* individuals, captured with the aggregation pheromone in the Colombian Pacific region. The molecular analysis of each single SAPW mitochondrial region Cytochrome c oxidase subunit I (CO1 or MT-CO1) allowed the allocation of each DNA sequencing to a species, demonstrating the chromatic variation for the species *R. palmarum* [31]. The male’s adults produce an aggregation pheromone that attracts both males and females (*E*-6-methyl-2-hepten-4-ol) [25]. The semiochemical is the primary control tool for this pest, participating in several mass-trapping opportunities with fresh and fermenting food lures. Much of the researchers’ concern has focused on the pure trapping ability of the devices [67,68], but studies on the cost/efficacy of attractant combinations [69,70] and the use of traps in ornamental palm polycultures [71,72,73,74,75] exist. Technology today suggests the usage of digital tools also to study the efficacy of traps [76].

## 3. Red Palm Weevil

The RPW, *R. ferrugineus* (Coleoptera: Curculionidae), is considered the worst plague of date [4,77] and urban [78] Canary palm trees worldwide, causing considerable damage to palm trees. It originates from tropical Asia, and in less than 40 years since 1972, it had spread from Sundaland to North Africa and the Mediterranean [9]. In the Americas, it first entered California in 2009 [12]. However, this invasive population was first incorrectly identified as the red palm weevil, *R. ferrugineus.* Further taxonomic classification confirmed that another species, *Rhynchophorus vulneratus* (Panzer, 1798), was responsible for the infestation of date palms in Laguna Beach, California, United States [22,79]. In 2008–2009, the RPW thrived on the islands of the American Caribbean, Curaçao, and Aruba [10].

Brazil considers *R. ferrugineus* an A1 quarantine pest for the country [80] with a considerable risk and potential threat for the Brazilian territory invasion. The risk for the commercial coconut and oil palm plantations increases due to the presence of the RPW in the Antilles (Aruba and Curaçao) [2,10] just at 64 km from the Venezuelan coast. Therefore, once the RPW invades these relevant crops, it could cause severe damage, significantly impacting Brazilian agriculture and native or commercial palm plantations in South America [17]. There are several review papers on the RPW, and the knowledge on the species is based on its impact on a more diversified array of environments and infested plants [22], eliciting the need for worldwide prediction modeling [5] and a rush for actual and future sustainable IPM control strategies also in just-invaded countries [14,16,81,82,83,84].

### Biology

*Rynchophorus ferrugineus* completes its life cycle in palms in approximately 80 days; the pest may develop up to four broods per year, with up to 3 months of adult longevity. All the instars live associated with bacteria and other organisms [53,54,85,86,87]. The holometabolic life cycle consists of the expected sequence of egg, larvae, pupae, and adult [9,28] (Table 1). Wattanapongsiri [15] has also reported on *Rhynchophorus* spp. biology in much of the stabilized literature.

Eggs: Females of *R. ferrugineus* oviposit for 8–10 weeks, laying an average of 250 eggs and up to 760 eggs per female during the reproductive life. The eggs are creamy-white, elongate-ovoid, slightly arched, measuring about 0.98 per 2.96 mm, and left in soft palm tissue. The egg stage lasts from 3 to 5 days.

Larvae: The final *R. ferrugineus* larvae (AKA grubs) are 36–46 mm long, eucephalous, apodous and “C”-shaped, and creamy-white if still feeding but orange in post-feeding, with the larvae instars lasting from 40 to 60 days. Larvae mine tunnels that are up one meter and a half long into palm stipes. Late damage sighting requires a complex pest management strategy, with the pest being practically undetectable until the strikes to the host plant are about lethal.

Pupae: The pupae *R. ferrugineus* are orange-yellow and remain housed within their 50–96 mm long pupal case that larvae set with palm fibers. The pupal stage lasts from 20 to 25 days.

Adults: An RPW adult male measures between 19 and 42 mm in length, the female between 26 and 40 mm, and rostrum features may serve to discriminate between male and female: the male snout is straight, stout, and shows series of stout seta set as in a brush on the front-clypeal head region, while the female rostrum is slender, devoid of seta, and slightly dorso-ventrally arched [83]. It has a typical adult chromatic polymorphism shown in the field [9,15]. The RPW is a highly aggressive pest that has been invading new niches worldwide [6]. The weevil is well active in high-temperature conditions between 26 and 30 °C and undergoes dormancy below 18.5 °C [82]. *Rhynchophorus ferrugineus* and *R. vulneratus* aggregate to a blend of two composites in pheromones produced by males, characterized as a “4-ethyl-5-nonanol” alkali and a “4-methyl-5-nonanone” ketone [22], but some field experiments showed an increment in the attraction for a stereoisomeric mixture (4*S*, 5*S*)-4-metilnonan-5-ol [24].

## 4. Damage to the Host Plant

Both *Rhynchophorus palmarum* and *R. ferrugineus* cause severe damage to the host plant. With any efficient control of these palm borers, they lead the plant to death and consequential damages. These insects feed on the sensitive tissue at the top of the palm, the larvae produce extensive galleries in the palm stipe, plants lose their ability to absorb nutrients, and the leaves turn yellow, hang, and fall [4,15,28].

In the American continent, the occurrence of *R. palmarum* causes considerable damage in sugar cane (*Saccharum officinarum* L.) [29], banana (*Musa sapientum* L.) [59], papaya (*Carica papaya* L.), and pineapple (*Ananas comosus* L.) [57] when feeding on sweet plant parts.

In Brazil, SAPW causes damage to coconut plantations (*Cocos nucifera* L.) and oil palm (*Elaeis guineensis* Jacq.) [1,35]. The Black Weevil transmits the *Bursaphelencus cocophilus* (Cobb) Baujard (Parasitaphelenchidae), the causal agent of the “Red Ring” or “Anillo Rojo,” whose name originated from the characteristic red belt always present in the stipe of diseased palms [26,61]. Red Ring external symptoms are present in the leaves, which turn yellow from the tip of the leaflets to the base of the rachis and then turn brown. Vector insect management currently achieves the red ring disease control, but there is no efficient method for controlling the nematode once it enters the palm [62]. RPW has known symbiotic nematodes such as *Mononchoides macrospiculum* (Nematoda: Neodiplogastridae) and *Teraorhabditis synpapillata* Sudhaus, 1985 (Nematoda: Rhabditidae) with a possible role in biocontrol [86,87]. The palms *Phoenix canariensis* Hort. ex Chabaud and *Phoenix dactylifera* L. are strongly susceptible to RPW. Significant damages appear in *P. canariensis* as a green crown drop-down in the shape of an “open umbrella” or as leaf wilt in *P. dactylifera*. Injuries become eventually visible only long after the palm has become infested. Unlike the infestation of *R. palmarum* that causes the red ring by *B. coccophilus*, the meristematic tissues of *P. canariensis* and *P. dactylifera* suffer infections due to symbiotic bacteria [53,54] and fungi that the RPW females lay by contaminated eggs.

Infested *P. canariensis* and *P. dactylifera* tissues root to a melted, hot fermenting matter with an acute alcohol-acid smell due to infection by *Serratia* spp., other bacteria, and yeasts that belong to *Candida* and *Hyphopichia* [85]. Such a guild of microorganisms enforces the pests’ fitness and creates a protective environment in infestation foci. In addition, in high-density infestations, the larvae burrowing and chewing the palm stipes produce audible sounds [88].

The control of the *R. palmarum* and *R. ferrugineus* has low efficiency in reducing damages, mainly due to the cryptic habit of its larvae inside the trunk [89]. Unexposed larvae can also eventually better survive the application of insecticides that badly impact humans and the environment. Cultural pest control consisting of timely uprooting and burning infested trees can reduce infestation but is often applied too late for use and has an environmental impact by greenhouse gas emissions [2,90].

## 5. *Rhynchophorus* spp. Pheromone and Other Attractive Sources

Behavioral control using aggregation pheromones enforced with food lures and ethyl acetate in mass trapping devices [3,43,70,74] propitiates the management, allowing a more confident use of insecticides.

### 5.1. Attractive Source for South American Palm Weevil

Attractive food sources associated with the aggregation pheromone improve the mass capture of *R. palmarum* by emulating the attraction for palm wound kairomones [70]. Moreover, the adults of *R. palmarum* use male-emitted semiochemicals as communication tools [25] to aggregate females and males at the “wound” spot. The aggregation pheromone alone is not as effective as needed in capturing adult *R. palmarum*, even if released at around 30 mg/day into the environment. Evidence suggests that the synergy among the plant kairomones, the pheromone, rotting plant parts such as sugar cane stalks, and ethyl acetate significantly captured more insects [70,73,91].

One study tested the attraction of *R. palmarum* to pieces of coconut palm, plantain, papaya, and pineapple. Banana was the most attractive lure peaking on the seventh day of fermentation [75]. The efficiency of modified traps associated with pheromone and bananas demonstrated one more opportunity for *R. palmarum* mass-trapping [73]. For the control of *R. palmarum,* the use of pieces of palm red-rings-infested stipe impregnated with insecticide also appears valuable despite the doubt raised by the possible nematode dispersion [26].

Attractiveness tests using the aggregation pheromone, 6-methyl-2 (E)-heptane-4-ol, associated with sugar cane stalks, pineapple fruit pieces, and six isolated volatile compounds of pineapple fruits, demonstrated that they do not show significant differences in the number of weevils captured [3]. A similar study [90] suggested that a combination of sugarcane with the aggregation pheromone increased the attraction of *R. palmarum* males and females toward traps, demonstrating the appearance of the synergy between these attractive sources. Adding ethyl acetate, sugar cane, and the aggregation of pheromone in traps exposed for three months had a significant effect on *R. palmarum* capture, demonstrating the synergy between the pheromone and other attractive substances. A similar synergism exists associating three days of pre-fermented baits for the attractiveness of *R. palmarum* [65,68]. The experiences are contradictory but suggest exploring the attractive potential of semiochemicals originating from fermenting plant material. The use of a new and inexpensive bait trap combination could offer significant benefits to producers and become a robust device to include in *R. palmarum* IPM [70,71]. Innovation in trap design and technology [69,76,92] may also play a role if novelties will well-embed management opportunities in local palm orchards.

### 5.2. Attractive Source for Red Palm Weevil

*Rhynchophorus ferrugineus* detection and management experience in different countries suggests that monitoring and ferrugineol-lured mass trapping are the most effective methods. The control method widely works in other countries of Asia and Europe [9,93]. Several studies, including the evaluation of stump traps, ethyl acetate as a lure synergist, kairomones, food baits, and yeast [94,95,96], exist to maximize the mass capture of *R. ferrugineus*. In addition, using black traps lured by pheromones added with a few dates can significantly increase the *R. ferrugineus* absolute collection number, minimizing the RPW impact and damage [94].

Electroantennography can verify the response of *R. ferrugineus* to volatile compounds [97], demonstrating that the finger palm esters (ethyl acetate, ethyl propionate, ethyl butyrate, and propyl butyrate) elicit strong responses in EAG trials but poor performance on the field. However, the synthetic mixture of pheromone with plant material and molasses appears ineffective in attracting and collecting *R. ferrugineus* adults [96].

**Figure 2:**
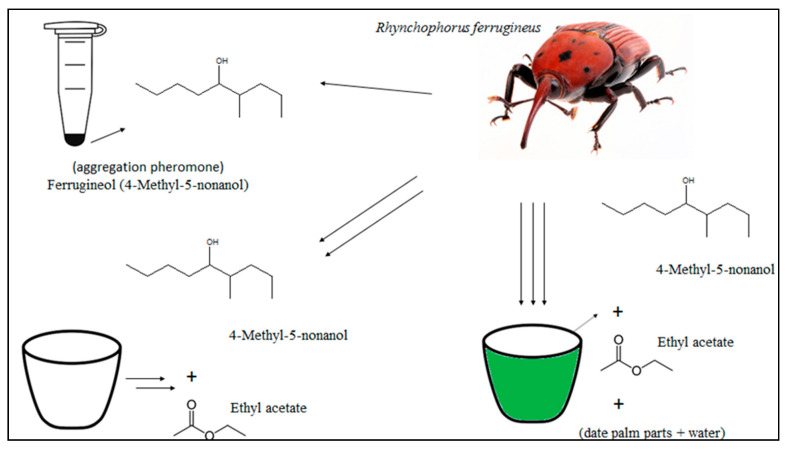
Pictorial scheme of the source used for *Rhynchophorus ferrugineus* luring.

Figure 2 shows the scheme of the aggregation pheromone, 4-methyl-5-nonanonol, and the synergy with ethyl acetate and parts of the palm tree. A blend of ferrugineol with plant kairomones or other palm tree volatiles effectively attracts adult RPW. A combination of synthetic lures, pheromone and ethyl acetate (released at 200–400 mg/day), and fermenting date palms parts in water increases the capture efficiency. The pheromone/food lure-based capture system is ecologically safer than insecticides for the current control of RPW infestations [98].

Studies [99] have explored the opportunity to increase the mass trapping of *R. ferrugineus* by stump traps, lures, lure synergists such as ethyl acetate, kairomones, food baits, and yeast. The stump trap lured more weevils than the tree trap did. The three tested pheromone lures were similar in attraction, but with ethyl acetate, Ferrolure^®^ TM lured more weevils than RHYFERTM^®^ did. Amongst the tested kairomones, acetic acid and ethyl acetate alone and together emerged as strong synergists of the lure. The date fruits, date palm stem pieces, and sugarcane pieces all attracted more weevils, but date fruits attracted significantly more adults in the presence of ethyl acetate. Date fruits attracted more weevils with yeast than alone, but overall, date fruits, yeast, and ethyl acetate together recorded the highest trap catches.

Regarding using traps with the aggregation pheromone associated with attractive sources, the experiences disbelieve the importance of defining a standardized system that makes it possible to attract and efficiently collect mass for *R. ferrugineus* [92]. The evaluation of three types of traps with pheromones PO28 Ferrolure and different VOCs (ethyl acetate, sugar cane cubes, and fruit pieces) obtained the best capture by the provided traps of the fermenting chamber and a cover with funnel [100].

Traps lured with a pheromone (ferrugineol), allomone (sugar beet juice), kairomone (ethyl acetate), and ester (ethyl propionate), each lure working alone or in combination, showed more females than males attraction. The best combination was in traps lured with ferrugineol + sugar beet juice + ethyl acetate + ethyl propionate than other chemical materials [95]. Improvements of the pheromones formulation technology, i.e., the use of pheromone in nanogels associated with dry funnel traps [44], disclose new opportunities.

## 6. Alternative for *Rhynchophorus palmarum* and *Rhynchophorus ferrugineus* Control

Further opportunities for efficient control-building strategies versus Red and South America Palm weevils rise from the use of parasitoids [55,56], entomopathogenic fungi [46,101,102], bacteria [53,66], nematodes as regulatory agents [86,89,103], volatile organic compounds [42,48,51], and biotechnological methods [38,41].

*Billaea rhynchophorae* (Blanchard) and *Billaea menezesi* (Guimarães) (Diptera: Tachinidae) [55,56] are indigenous larval parasitoids of *R. palmarum* in Brazil. A better knowledge of both Tachinidae lifecycle and host acceptation will tell if they can serve in the inundative biological control of all *Rhynchophorus*, pest species. Parasitoidism can join other control methods to enforce the management efficacy and minimize the actual *R. palmarum* impact [2,3].

Likewise, a study [55] suggested the importance of *Billaea* spp. introduction and mass release—not available at the time—in coconut and oil palm production areas waiting for the potential invasion of *R. ferrugineus*. By that time, *Billaea* targeted five different genera of palm beetles, proposing natural biocontrol in the state of Bahia-Brazil. However, we need accurate studies to establish economic mass multiplication techniques and the proper inclusion of Diptera in palm weevil IPM.

Among the entomopathogenic fungi, the *Beauveria bassiana* and *Metharizium anisopliae* are relevant for biological pest control of palm borers [104,105]. The fungi can induce mycosis in various niches, leading to epizootics [46,106]. Most of these fungi assemble in a complex of about 90 genera and 700 species [107] of soil inhabitant species responsible for approximately 80% of insect diseases occurring without propagule ingestion [108,109].

The infective process starts when spores germinate on the insect’s cuticle [49,109]. Chitinolytic enzyme secretion is the primary key to the virulence of the entomopathogenic fungi [107,108]. Fungi invade insects through the integument, provoking a systemic invasion in the hemocoel by releasing enzymes such as endoproteases, aminoproteases, lipases, esterases, and chitinases. The death of the insect follows about five days later [103,104,109].

**Figure 3:**
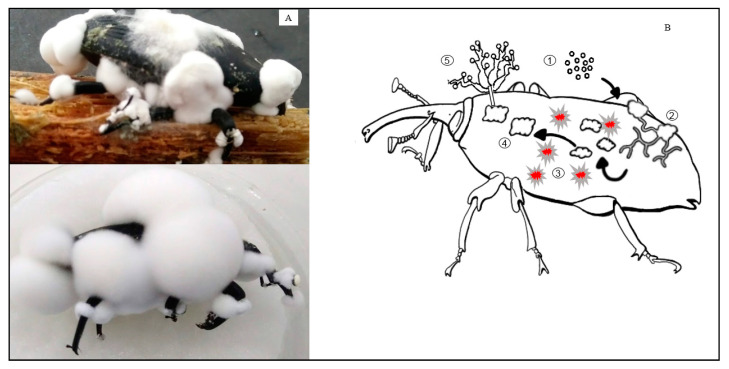
Schematic diagram of the infection process by *B. bassiana* after visiting a baiting and contaminating station. (**A**) Infection fungi process in the laboratory showing pathogenicity and mortality; (**B**) illustrative infection cycle depicted by *B. bassiana* in *R. palmarum*: (1) spore adhesion; (2) cuticle hydrolysis and breaking; (3) fungal cells change their cell structures in contact with hemolymph and secrete secondary metabolites; (4) switch to a yeast-like structure to colonize and kill the insect; and (5) sporulation.

Fungal infection (Figure 3) causes insect septicemia, a lethal disease, bacterial contamination, and rotting. The same microorganisms produce VOCs and other semiochemicals bearing information to the receiving organisms. VOCs are also media for interspecific communications ruling fungi–insect interactions [42,110]. Thus, VOCs secreted by fungi may represent a source of promising compounds for biocontrol and pest manipulation despite also having antifungal, insecticidal, attractive, or repellent properties [111]. Species of Coleoptera, Isoptera, Hemiptera, and Orthoptera, once they have detected specific VOCs produced by entomopathogenic fungi of the Hypocreales, change their behavior to avoid mycosis [112].

For the use in a control strategy, a study [47] referred to 22 organic compounds of *B. bassiana* capable of repelling *R. ferrugineus*. Evidence suggests using repellant as a “puller” associated with traps lured with aggregation pheromones for ornamental palms protection. For banana weevil (*Cosmopolites sordidus* Germar, 1824), management [48] suggests similar opportunities characterizing ninety-seven profiles of fungal VOCs from *B. bassiana* isolate Bb1TS11, *Metarhizium robertsii* isolate Mr4TS04, and *Pochonia chlamydosporia* (Goddard) isolate Pc123. In the experiences, the compounds 3-cyclohepten-1-one and 1,3-dimethoxybenzene show repellence in banana weevil. The insect–fungal interactions can be a new allelochemicals-based interaction of particular benefit for developing innovative strategies for pest management [45]. Volatile organic compounds combined with aggregation pheromones interplay in a push–pull method to control *R. ferrugineus* [113,114].

Another technique of great importance is the use of botanical oils as a sanitary control method versus insect pests [52,115] given the effect of botanical oils of *Melissa officinalis* L. (Lamiaceae), *Borago officinalis* (L.) (Boraginaceae), *Laurus nobilis* L. (Lauraceae), and *Carapichea ipecacuanha* (Brot.) L. Andersson (Rubiaceae) for the control of *R. ferrugineus*. The results showed that males of *R. ferrugineus* were more reactive to the four botanical oils than females were. Botanical oils remarkably reduce adult longevity, female fecundity, and eggs hatchability. The larvae showed different degrees of susceptibility to the four botanical oils.

The impacts of sesquiterpenes secondary plant metabolites, including farnesol, farnesyl acetate, Picrotoxin, β-caryophyllene, (+)—cedrol, nerolidol, (+)—nootkatone, and parthenolide, on *R. ferrugineus* are also puzzling [8]. Picrotoxin, farnesol, and farnesyl acetate are compounds most toxic to larvae of eight instar. Picrotoxin showed lethal effects and reduced food indexes, providing significant inductions of glutathione-S-transferase (GST) and cytochrome P450 gene expressions. These results may suggest using Picrotoxin as a promising biopesticide to control red weevil infestations, despite the difficulties of safely delivering such a venomous and reactive compound.

Molecular biology has provided some promises [39], eliciting interest in adopting biotechnological alternatives to pest control, mitigating the damage in different crop pests. The RNAi technique breaks the odor-binding protein, thus preventing the insects from tracking specific semiochemicals [38]. The interruption of the olfactory ability leads to reduced pheromones detection and suggests a promising alternative for *R. ferrugineus* [39,106,109]. The RNAi silencing of *R. ferrugineus* RferOBP1768 appears in EAG tests with a reduction in the weevil responses to the aggregation pheromone.

Among the technologies applied to detect insects that cause injuries to the tree trunks, there are devices capturing signals emitted by the larvae, identifying the infestation in the culture [24,37].

A protective and preventive IPM strategy [116] will considerably manage the pest population and damage, with every control method array that we intend to apply.

## 7. Future Challenges and Prospects

This review aimed to inform and will contribute to a better knowledge of palm weevil pest control. The focus here was on the efficacy of some attractive or repellant sources for weevil management, but we also considered secondary conventional or innovative control methods. The suggestion is to view all the *Rhynchophorus* and allied species as study model pests based on homogeneous bionomics. The advice also rises for the current alarm on *Dynamis borassi* in the New World [117,118].

The key tract of the palm weevil pestiferous attitude is the late evidence of the ongoing infestation. The tract unexpectedly assimilates palm weevil control to several other insect-borne plant pathogens such as *Xylella fastidiosa pauca* ST53 (Wells et al., 1987) [119], whose damages appear years after the first transmission. Unluckily, both the adversities will be managed better by preventive and protective strategies [116], with the critical factor in the timing of the control actions [120,121]. Volatile compounds experience suggests their use in proper combination [122] and with effective traps, preferably dry. Experiments have shown that male and female attraction depends not only on each component, volatiles compounds, pheromones, or kairomones, but also on a significant synergism among components [42,43,90].

The advances developed in recent decades in America for the control of *R. palmarum* shows promising results in expanding the management experience to a preventive and protective strategy against *Rhynchophorus ferrugineus*.

Effective pest mass rearing techniques [123] will enforce a palm weevil IPM strategy by controlling actions. The combination of control methods based on semiochemicals, contaminating stations, Diptera parasitoid mass release, and further natural enemies’ enrichment promises effective management options. The assemblage is advantageous with the VOCs repellant properties, eventually pulling the parasitoids versus the uncontaminated target pests only.

The basis is wide enough for research and technology transfer that will quickly turn to sustainability and environmentally safe control tools if the stakeholders learn the IPM rationale lesson [124].

In 2018, the Brazilian Ministry of Agriculture and Supply [80] already registered the *Rhynchophorus ferrugineus* as an A1 quarantine pest for Brazil, making the first relevant step to consider the opportunity for combined effective control of SAPW and RPW.

Since 2018, the Brazilian EMBRAPA Tabuleiros Costeiros, the Natural Resources (LPqRN) of the Federal University of Alagoas (UFAL, Brazil), and the Colombian Corporation for Agricultural Research (Agrosavia) have run the RPW control challenge in SAPW-invaded territories. The following steps will suggest control strategies tuning, enlightening, in New World palm weevils, the factors that triggered the RPW population [125] from endemism to outbreak.

## Figures and Tables

**Table 1:** *Rhynchophorus palmarum* and *Rhynchophorus ferrugineus* quick card.

	*Rhynchophorus palmarum*			*Rhynchophorus ferrugineus*			
Phase	Measure (mm)	Duration (Days)	Colour	Measure (mm)	Duration (Days)	Colour	Source
Eggs	25 × 10	2–5	White	9 × 29	3–5	white	[15,21]
Larve	75	45–70	Yellowish	36–46	40–60	yellowish	[15,21]
Pupae	80–100	25–45	Yellowish	50–95	20–25	yellowish	[15,21]
Adults	35–60	60–95	black and reddish atypical chromatic natural polymorphism	(♂): 19–42	60–95	rust red, typical chromatic natural polymorphism	[9,15,31]
				(♀): 26–40			
Total cycle	132 to 215 days			82 days			[15,21]

## Data Availability

The data presented in this study are available in article.
